# Single-cell genomics in plants: current state, future directions, and hurdles to overcome

**DOI:** 10.1093/plphys/kiab478

**Published:** 2021-10-18

**Authors:** Josh T Cuperus

**Affiliations:** Department of Genome Sciences, University of Washington, Seattle, Washington, USA

## Abstract

Single-cell genomics has the potential to revolutionize the study of plant development and tissue-specific responses to environmental stimuli by revealing heretofore unknown players and gene regulatory processes. Here, I focus on the current state of single-cell genomics in plants, emerging technologies and applications, in addition to outlining possible future directions for experiments. I describe approaches to enable cheaper and larger experiments and technologies to measure multiple types of molecules to better model and understand cell types and their different states and trajectories throughout development. Lastly, I discuss the inherent limitations of single-cell studies and the technological hurdles that need to be overcome to widely apply single-cell genomics in crops to generate the greatest possible knowledge gain.

## Teaser

With single-cell genomics having the potential to revolutionize the study of plant development, we address the current state, future directions, and hurdles to overcome.

## Introduction

In many ways, the promise of single-cell genomics—so well demonstrated in animal and human research—has finally arrived in plants. In 2019, five publications, appearing almost simultaneously, characterized gene expression in single Arabidopsis (*Arabidopsis thaliana*) root cells (Denyer et al., 2019; Jean-Baptiste et al., 2019; Ryu et al., 2019; Shulse et al., 2019; Zhang et al., 2019). The Arabidopsis root was the perfect system for these proof-of-principle studies. With few cell types and extensive existing knowledge on cell-type-specific expression and developmental trajectories (Brady et al., 2007), these studies were able to build on the known “ground truth” of cell-type proportions and spatial and temporal expression patterns. Since then, single-cell genomics has been applied to other plants, including crops like maize (Kim et al., 2021; Marand et al., 2021; Ortiz-Ramirez et al., 2021) and rice (Wang et al., 2020; Liu et al., 2021), and has contributed to the discovery of previously unknown biology, such as genes acting in lateral root development (Gala et al., 2021), or regulating root hair density in response to phosphate dosage (Wendrich et al., 2020). All studies published to date highlight the promise of single-cell genomics for fully understanding plant gene regulation; however, they also highlight the existing challenges before this promise can be fully realized. The first challenge is clear: we need to analyze many more cells to capture all the cell types and their various developmental states in large crops like maize.

## We need more cells: some biological questions require more cells 

The first droplet-based single-cell studies examined only a few thousand root cells (Denyer et al., 2019, 4,727 cells; Jean-Baptiste et al., 2019, 3,121 cells; Ryu et al., 2019, 7,522 cells; Shulse et al., 2019, 12,198 cells; Zhang et al., 2019, 7,695 cells). In stark contrast, single-cell studies in animal systems often rely on tens of thousands if not millions of cells to draw conclusions—for example, two million cells were assayed in a 2019 study of mouse embryos (Cao et al., 2019), 50,000 cells in a planarian single-cell transcriptome study (Fincher et al., 2018), and 80,000 cells in the *Caenorhabditis* *elegans* embryogenesis atlas (Packer et al., 2019). Although more does not always equate better, there is a strong argument for this to be true in single-cell genomics. Combining the 2019 Arabidopsis root single-cell datasets boosted cell numbers to about 50,000 and filled in developmental trajectories for some cell layers (McFaline-Figueroa et al., 2020). Following this approach, others subsequently integrated their newly generated, larger datasets with this existing data (for a total of approximately 96,000 cells), resulting in the largest, most complete analysis of Arabidopsis root development to date (Shahan et al., 2020). Although the current cell numbers may have sufficed to study the exceedingly well-characterized Arabidopsis root, as the field moves to profiling more complex tissues of less well-characterized, larger plants, the ability to profile a greater number of cells will be critical for statistical rigor in interpreting the results. This is particularly true for capturing developmental transitions or specific cell states which will be present only in a few cells of a given cell type. It is worth noting that the ability to profile large numbers of cells negates the need for sampling different developmental stages because developmental trajectories of different cell layers, tissues, and organs can be readily reconstructed from high-quality data (Fincher et al., 2018).

Large cell numbers and data integration are critical not only for comparing wild-type cell layers and tissues but also for detecting differences in cell-type proportions or cell-type-specific expression patterns in mutants (Ryu et al., 2019; Shahan et al., 2020). For example, by profiling mutants for the genes SCARECROW and SHORTROOT and comparing them to wild-type, Shahan et al. provide evidence for an alternative pathway that defines endodermal identity in the absence of SCARECROW but requires SHORTROOT function (Shahan et al., 2020). The increase in knowledge gain made possible by larger cell numbers is also well illustrated in a recent maize study that assayed more than 50,000 nuclei for chromatin accessibility (Marand et al., 2021). Chromatin accessibility was captured from nuclei representing several tissues and stages of development, and tissue- and stage-specific accessible sites were integrated with other features such as genome organization and genetic diversity. As a result, the authors built a comprehensive framework in order to describe and understand gene regulation and its role in phenotypic diversity and domestication. One finding points toward several transcription factor families and their binding sites as acting in a fashion similar to the transcription factor CCCTC-binding factor (CTCF) and its binding sites in metazoans, which define the borders of chromatin loops with CTCF blocking the interaction between enhancers and promoters. Plant genomes do not contain CTCF homologs but surely its functional equivalent must exist to ensure the precision of plant gene expression and its dynamics in development and environmental responses. Marand et al. offer a first glimpse of possible functional equivalents, a finding that will undoubtedly stimulate further research.

Although more recent plant studies include increased numbers of cells, the current cost per nucleus is still very high, keeping the technology out of reach for many plant research groups. Commercial technologies such as the droplet-based method offered by 10× Genomics cost about $1.00 per cell, not including labor or downstream analysis. There are other, cheaper technologies developed in animal systems that could be applied to plants: single-cell combinatorial indexing, split-seq, and scifi-RNA-seq, among others (Cao et al., 2017; Rosenberg et al., 2018; Datlinger et al., 2019). Neither combinatorial indexing nor split-seq relies on a droplet-based approach; rather, they are based on several rounds of marking and mixing cells in a multiplexed format. As a result, each cell’s transcripts and/or accessible sites carry a unique combination of barcodes that can be attributed back to that cell. This approach has been applied extensively in mammalian cell culture experiments as well as to whole animals and bacterial cultures (Rosenberg et al., 2018; Cao et al., 2019; McFaline-Figueroa et al., 2019; Srivatsan et al., 2020; Kuchina et al., 2021).

Methods like “hashing” or MULTI-seq are multiplexing methods to mark cells (Stoeckius et al., 2017; Shin et al., 2019; McGinnis et al., 2019b; Gehring et al., 2020; Fang et al., 2021). In its simplest form, hashing relies on DNA oligos that mimic polyadenylated RNAs, but with well-specific barcodes, which enables large-scale assays of cells exposed to many different conditions, for example, drug concentrations (Srivatsan et al., 2020). Hashing could be used to increase the throughput of droplet-based methods by allowing to overload droplets with multiple cells or nuclei. The recently published scifi method uses a similar multiplexing approach, combining one-step combinatorial preindexing of single-cell transcriptomes with subsequent single-cell RNA-seq using commercially available droplet microfluidics. Similar to hashing, the preindexing step allows the loading of multiple cells per droplet, which increases the throughput of droplet-based single-cell RNA-seq up to 15-fold, enabling the multiplexing of many samples in a single scifi-RNA-seq experiment (Datlinger et al., 2019). Both hashing and scifi could make the 10× Genomics platform more affordable for plant researchers; however, developing plant-specific methods that do not rely on proprietary kits and instrumentation would further reduce costs.

There are drawbacks to increase the number of cells through combinations of barcodes: the higher the number of combinations (e.g. 2 versus 3 in combinatorial indexing), the fewer transcripts per cells are typically captured. The more cells are included in an experiment, the higher the incidence of barcode collision (i.e. two cells carrying the same set of barcodes) and thus “wasted” sequencing, which requires computational follow-up steps to remove suspect cells (Wolock et al., 2019; DePasquale et al., 2019; McGinnis et al., 2019a). These methods commonly rely on simulating doublets and identify real doublets by similarity, allowing for their removal from the analysis. Multiplexing based on natural variation is another strategy to increase throughput and simultaneously detect doublets effectively (Kang et al., 2018).

More recent single-cell genomics studies often profile nuclei rather than whole cells, presumably capturing a greater number of newly transcribed molecules. Although nuclei-based methods capture fewer molecules, these may be more biologically informative (Bakken et al., 2018; Farmer et al., 2021). Researchers are rightly concerned with the minimum requirements for cell numbers and number of molecules measured. At this point, however, it is challenging to formulate general guidelines, especially since these requirements critically depend on the experimental question asked. For example, if the scientific question concerns the relative size of a certain cell population among mutants, measuring a few, cell-type-specific transcripts can suffice. If the scientific question focuses on differential cell states or developmental transitions, many more cells and measured transcripts are needed for a statistically rigorous, meaningful analysis.

## Capturing low-abundance cells

During development, heterogeneity in gene expression and ultimately different cell fates arise from a population of formerly identical cells. Many researchers are keen to capture the initial emergence of this regulatory heterogeneity in development, which likely occurs only in a small number of cells. Unfortunately, these cells will represent only a tiny fraction of the cells collected in a typical single-cell experiment, limiting statistical power. Because the costs per cell are still very high, simply expanding the number of cells is not the best answer. As an alternative, several groups have sought to enrich low-abundance cell types. Their strategies included careful tissue selection, for example, focusing on young seedlings, increased digestion of plant tissue to enrich for vasculature cells, tissue dissection, or physical removal of overrepresented cells (Bezrutczyk et al., 2021; Gala et al., 2021; Kim et al., 2021). These strategies met with varying success: mesophyll cells still accounted for ∼75% of all assayed cells in Arabidopsis leaves (Bezrutczyk et al., 2021) and 98% in maize leaves (Bezrutczyk et al., 2021). With as few as approximately 50 cells, Bezrutczyk et al. still found many differentially expressed genes, subsequently validated with in situ hybridization. Similarly, Gala et al. relied on only 167 lateral root primordia (LRP) cells out of nearly 7,000 sequenced cells to identify approximately 800 differentially expressed, LRP-specific genes (Gala et al., 2021). Several of the selected top candidate genes influenced lateral root development when their expression was manipulated in validation experiments. Other groups have tackled low abundance cell types in even higher resolution, focusing on LRP in Arabidopsis (Serrano-Ron et al., 2021), shoot-borne-root meristems in tomato (Omary et al., 2020), inflorescence tissue in maize (Xu et al., 2021), and phloem root cells in Arabidopsis (Roszak et al., 2021).

Although these examples give us confidence that important knowledge can be gleaned even from a small number of cells, efficient enrichment methods are a high priority in the field. One proven strategy is fluorescence-activated cell sorting of either protoplasted cells or nuclei (Brady et al., 2007); a similar strategy relies on the capture of biotinylated nuclei (Deal and Henikoff, 2011). Both methods require prior knowledge of genes with cell-type or condition-specific expression; they also rely on transgenic lines that carry the respective constructs marking a particular cell type. Ultimately, the solution to capture low abundance cell types in crops will have to involve both: The development of innovative enrichment strategies coupled with the adaptation of sequencing methods that allow the analysis of millions of cells at a reasonable price.

## Cell-type annotation without the deep prior knowledge gathered from marker lines

A major challenge of all single-cell genomics studies is the correct annotation of cell types. It is no co-incidence that the first plant single-cell RNA-seq studies focused on the Arabidopsis root (Denyer et al., 2019; Jean-Baptiste et al., 2019; Ryu et al., 2019; Shulse et al., 2019; Zhang et al., 2019), and that in animals *C. elegans* with its exceedingly well-characterized invariant cell lineage (Sulston et al., 1983) served as a model for organismal single-cell genomics (Cao et al., 2017; Packer et al., 2019). Even in maize with its good annotation of certain tissue-specific expression patterns, cell type annotation remains a challenge. Marand et al. used extensive literature searches to identify the number of marker genes necessary to call a cluster reliably (Marand et al., 2021).

The initial Arabidopsis single-cell studies critically relied on root cell-type-specific gene expression data derived from the fluorescent marker lines that researchers had previously generated (Birnbaum et al., 2003). These resources are less available for shoot cell layers, especially throughout development. Such marker lines are even less common for most crops or really any other plant species on earth. A recent study ingeniously tried to overcome this limitation. Ortiz-Ramirez et al. relied on fluorescent dyes and an endodermis marker line to capture different maize cell types. Specifically, they used dyes with different abilities to penetrate cell layers to sort exterior and interior cell layers of maize root tips followed by RNA-seq. This approach facilitated the identification of cell clusters in their single-cell RNA-seq experiments, allowing them to confidently identify 16 distinct clusters at a sub-tissue level (Ortiz-Ramirez et al., 2021). The dye-penetrance method can easily be applied to other plants; however, is likely limited to root tips in its current form.

In order to overcome the challenge of cell-type annotation, animal researchers have developed computational approaches to predict cell types in single-cell space based on previous experiments. Although this approach has not yet been applied outside of mammalian systems, programs like Garnet, clustifyR, scClassify, scNym, and scCATCH can predict cell types for the same types of data from the same organism with high reproducibility (Pliner et al., 2019; Fu et al., 2020; Kimmel and Kelley, 2020; Lin et al., 2020; Shao et al., 2020). I envision a pan-classifier that builds on existing single-cell studies across several plant species, and includes homolog information to predict cell types in single-cell experiments in additional plant species without the reliance on extensive marker genes and tissue level bulk RNA-seq. There is evidence from animal systems for this to work: for Garnet, cell-type annotation across species was remarkably accurate when using mouse data to classify human single cells (Pliner et al., 2019). Using single-cell data from mouse lung cells, over 92% of alveolar, B cells, T cells, epithelial (ciliated) cells, endothelial cells, and fibroblasts were accurately assigned in a human lung tumor.

Similar approaches in plants are faced with the challenge of deep divergence in the plant kingdom. Monocots and dicots are far more diverged than mouse and human, and even within dicots, many tissues do not share cell-type-specific expression patterns (Kajala et al., 2021). Nevertheless, other aspects of gene regulation such as transcription factors and their binding motifs are conserved across plants. The potential for classifying cell types in less-well characterized plants on the basis of existing single-cell data from Arabidopsis is well illustrated by a recent study that tested several of the 111 identified marker genes for trichoblast cells in five other plant species (Yan et al., 2020). As the efficacy of machine learning relies critically on data volume, the results will only improve as more and more datasets become available.

## Using the power of long reads to gain high-resolution knowledge

Long-read sequencing is getting more affordable and more accurate (Wenger et al., 2019), making it possible to apply it to single cells. There are already several groups that have successfully applied long-read sequencing to different systems (Gupta et al., 2018; Singh et al., 2019; Lebrigand et al., 2020; Volden and Vollmers, 2020; Hård et al., 2021; Long et al., 2021). Long et al. used a combination of Illumina and Nanopore sequencing to assay transcriptomes in Arabidopsis roots at single-cell resolution, enabling them to identify cell-type-specific splice forms (Long et al., 2021). This is clearly an important step forward to understanding plant development and plant responses to environmental stimuli because alternative splicing is so prevalent in plants.

There are also new technologies that rely on long-read technology to assay chromatin occupancy by nucleosomes and transcription factors (Abdulhay et al., 2020; Stergachis et al., 2020). Because both Nanopore and PacBio can detect DNA methylation, these methods rely on methylating accessible DNA and reading out the resulting patterns on 10 kb or larger DNA molecules. In this way, accessible regions, transcription factor footprints, and nucleosomes can be assayed in context with one another, which could help to overcome the sparseness of single-cell ATAC data. Although these methods have not yet been applied to single cells or even just to plants, I predict that it is only a matter of time when this will be attempted.

## Co-assays for maximal knowledge gain

Several software packages allow researchers to computationally combine different single-cell measurements. For example, Dorrity et al. co-embedded existing single-cell RNA-seq from Arabidopsis roots with their newly generated ATAC-seq data from young roots (Dorrity et al., 2020). This approach allowed the authors to annotate their cells with the much richer features that can be gathered from gene expression data, including scores for cell cycle state, endoreduplication, and developmental progression. They were also able to associate the increase of chromatin accessibility at sites containing a binding motif for a certain transcription factor family with the increase in expression of a specific member of this transcription factor family. In short, they were able to make inferences about specific regulatory events at individual loci, something the field has struggled to achieve due to the generally poor association of chromatin accessibility and gene expression.

In animals, combinatorial indexing has been used to co-capture chromatin accessibility and gene expression; however, this co-assay reduces power for both measures because many fewer molecules are captured (Cao et al., 2018). Although I am not aware of any publications or preprints applying them yet, commercial platforms have begun to offer droplet-based co-assays. Notably, the published co-assays of accessibility and gene expression continue to observe the weak correlation between chromatin accessibility and gene expression that we and others have attributed to regulatory complexity (Brady et al., 2011; Gaudinier et al., 2011; MacNeil et al., 2015; Fuxman Bass et al., 2016; Alexandre et al., 2017; Cao et al., 2018; Chen et al., 2019; Dorrity et al., 2020). A weak correlation is even observed when associating only the accessibility of promoters of differentially expressed genes with their expression levels in mouse cerebral cortex clusters (*r* = 0.34 on average; Chen et al., 2019). Co-assays of accessibility and gene expression in plants with their more plastic development may help facilitate the resolution of the ongoing debate whether to attribute the low correlation between accessibility and expression to regulatory complexity, technical limitations, or a combination of both.

Plants derive much of their unsurpassed capacity for phenotypic plasticity from their vast universe of small RNAs. Although there have been a few small-scale attempts to measure both small RNAs and messenger RNA in mammalian cell culture (Wang et al., 2019), this type of co-assay really needs to be applied in plants to gain important biological insights. Capturing the regulatory small RNAs in plants in conjunction with the mRNAs and genes they regulate will enable us to better understand gene regulation at a single-cell level. In stark contrast to animal systems, small RNAs in plants rely on full or near full sequence alignment to target sites, which readily enables target predictions. This technology will likely be pioneered in Arabidopsis or maize due to the wealth of existing tools.

Another, only somewhat futuristic, plant-specific application of single-cell genomics would be the potential to multiplex experiments by exploring the effects of hormones, herbicides, temperature, or fertilizers on seedlings, possibly including mutant collections. I envision these experiments with very small plant species, such as duckweed, or with young seedlings. Although comparable human single-cell research can rely on multiplexing cell lines, plant researchers are able to work within the organismal context that matters for responses to genetic or environmental perturbations.

## To what end?

The hope and promise of single-cell genomics are that someday we will fully understand gene expression in order to predict and manipulate gene expression in future crops, using either natural variants or targeted genome editing. As traditional breeding becomes too slow to accommodate the rapid changes in the environmental conditions around us, this knowledge is critical for obtaining food and energy security.

**Advances**

- Single-cell genomics has moved beyond model systems into crops, including maize and rice, with new understanding of tissue-level expression and accessibility.
- New technologies in other systems give us hope that plant biologists can both increase the number of cells assayed in single-cell experiments and decrease the cost to assay these cells.
- Software is emerging to help cluster and annotate cell types without extensive prior knowledge or marker genes for that tissue.

**Outstanding questions**

- Will single-cell genomics help us resolve the relationship between accessibility and gene expression?
- How will we harness the underlying information we gather from single-cell experiments to manipulate crops for better outcomes?
- What emerging technologies can we best apply to plant systems with meaningful outcomes?

## Notes

The author responsible for distribution of materials integral to the findings presented in this article in accordance with the policy described in the Instructions for Authors (https://academic.oup.com/plphys/pages/general-instructions) is Josh T. Cuperus (cuperusj@uw.edu).
